# Methods for quantifying the heterogeneity of psychopathology

**DOI:** 10.1186/s12888-023-05377-5

**Published:** 2023-11-30

**Authors:** John F. Buss, Ashley L. Watts, Lorenzo Lorenzo-Luaces

**Affiliations:** 1 Department of Psychological and Brain Sciences, Indiana University, Bloomington, IN 47405 USA; 2 Department of Psychology, Vanderbilt University, Nashville, TN 37420 USA

**Keywords:** Depression, Classification, Melancholia, Atypical

## Abstract

**Objectives:**

Specifiers for a major depressive disorder (MDE) are supposed to reduce diagnostic heterogeneity. However, recent literature challenges the idea that the atypical and melancholic specifiers identify more homogenous or coherent subgroups. We introduce the usage of distance metrics to characterize symptom heterogeneity. We attempt to replicate prior findings and explore whether symptom heterogeneity is reduced using specifier subgroups.

**Methods:**

We used data derived from the National Epidemiological Survey on Alcohol and Related Conditions (NESARC Wave I; *N* = 5,749) and the Sequenced Treatment Alternatives to Relieve Depression study (STAR*D; *N* = 2,498). We computed Hamming and Manhattan distances from study participants’ unique symptom profiles. Distances were standardized from 0-1 and compared by their within- and between-group similarities to their non-specifier counterparts for the melancholic and atypical specifiers.

**Results:**

There was no evidence of statistically significant differences in heterogeneity for specifier (i.e., melancholic or atypical) vs. non-specifier designations (i.e., non-melancholic vs. non-atypical).

**Conclusion:**

Replicating prior work, melancholic and atypical depression specifiers appear to have limited utility in reducing heterogeneity. The current study does not support the claim that specifiers create more coherent subgroups as operationalized by similarity in the number of symptoms and their severity. Distance metrics are useful for quantifying symptom heterogeneity.

## Background

A major depressive episode (MDE) involves a combination of symptoms [1]. To meet the symptom criteria for an MDE, an individual must present with five of nine possible symptoms for two weeks, and at least one symptom must be sadness or anhedonia. Some symptoms of an MDE can be met by reporting qualitatively different complaints (e.g., symptom six can be met by reporting either fatigue or loss of energy). Other symptoms can be met by reporting complaints that differ in severity (e.g., symptom nine can be met by reporting recurrent thoughts of death or by attempting to commit suicide). Still, some symptoms represent opposites (e.g., symptom five can be met by psychomotor agitation, but it can also be met by psychomotor retardation and symptom four can be met by insomnia or hypersomnia). Using polythetic criteria for an MDE leads to highly heterogeneous symptom presentations to the point that two individuals with an MDE may not share a single symptom [2, 3]. Researchers often quantify diagnostic heterogeneity in symptom presentations by counting the number of symptom combinations possible and reported, which we refer to as “symptom profile categories.” For example, in the Sequenced Treatment Alternatives to Relieve Depression (STAR*D) sample of *N* = 3,703 outpatients, there were 1,030 depression symptom profile categories [2, 4]. Proponents of both dimensional [5, 6] and network theory approaches [7] have used the high number of symptom profile categories as a rationale for new approaches to conceptualizing psychopathology.

The Diagnostic and Statistical Manual for Mental Disorders 5th edition (DSM-5) uses specifiers for depression and other diagnostic subgroups (APA, 2013). According to the DSM-5, individuals who share specifier features are more similar to each other than individuals who do not share the specifier features and thus create “more homogeneous” subgroups. However, recent research suggests that specifiers do not create more homogeneous subgroups [8, 9]. Specifiers for MDE subgroups classify individuals by adding polythetic features to the DSM criteria. Combinatorics thus suggest that the DSM specifier subgrouping system may create more *heterogeneous* subgroups [9]. In an analysis of heterogeneity in the melancholic and atypical specifiers in STAR*D, reductions in heterogeneity when comparing subgroups of individuals that met criteria for a specifier (e.g., melancholic) vs. those that did not (e.g., non-melancholic features) were not significant. Any apparent reductions in heterogeneity appeared driven by smaller sample sizes in the specifier subgroups than would be expected by chance [8].

Although the findings of Lorenzo-Luaces et al. [8] are consistent with the combinatorics logic previously presented by Fried et al., they have not yet been replicated. Moreover, Lorenzo-Luaces et al. quantified heterogeneity using symptom profile categories. Symptom profile categories are a very strict approach where two individuals are considered to have heterogeneous symptom presentations if they differ on only one symptom. This strict approach has been used in several studies [3, 10], but it treats heterogeneity as a binary variable (i.e., individuals are either the same or they are not). This categorical approach is inconsistent with the concept of assessing psychopathology along a continuum [11]. A strict binary approach acts as a very rough measure of heterogeneity. It also imposes a high bar for proving specifiers useful because all it takes is one dissimilar symptom for individuals to be considered “different.”

Given the limitations associated with previous studies, we sought to replicate the findings of Lorenzo-Luaces et al. [8], namely that the specifier subgroups do not reduce heterogeneity, using a large nationally-representative sample of adults (*n* = 5,749). Rather than rely on a simple, binary metric that indicates whether or not diagnostic combinations were 100% identical, we used distance metrics in N-dimensional space to quantify the extent of heterogeneity on a continuum. Specifically, we used the Hamming and Manhattan distances to characterize the relative similarity of symptom profile categories. We hypothesized that atypical and melancholic specifiers would not reduce heterogeneity relative to the non-atypical and non-melancholic groups. Additionally, we reanalyzed the STAR*D data (*n* = 2,498) to explore whether previous results were driven by the fact heterogeneity was operationalized categorically as opposed to continuously. We refer to a *diagnostic combination* as any set of symptoms defined in the DSM-5, such that an individual meets criteria for an MDE. *Coherence* is the amount of within-group homogeneity, where greater coherence indicates greater homogeneity within a given subgroup. In contrast to coherence, we use the term *distance* to refer to the degree of heterogeneity as measured by distance metrics. We define *differentiation* as the ability of subgroup diagnostic criteria to define subgroups with markedly different diagnostic combinations.

## Methods

### NESARC

We analyzed the public-access dataset from the NIAAA-supported National Epidemiologic Survey on Alcohol and Related Conditions (NESARC), Wave I study [12]. The NESARC was a nationally representative study of adults 18 years or older (*N* = 43,093) who were interviewed face-to-face using the Alcohol Use Disorder and Associated Disabilities Interview Schedule-DSM-IV (AUDADIS-IV). The NESARC sampled sociodemographic subgroups to ensure that the sample sufficiently represented the US population (e.g., Hispanic, Non-Hispanic Black, and young adults) with a response rate of 81%. From the total number of respondents, 7,839 met criteria for an MDE in their lifetimes. Participants were excluded from our analyses if they A) met criteria for mania or hypomania (*n* = 725), or B) their worst episode experienced was deemed illness or substance-induced (*n* = 715). After exclusion criteria were applied, 6,448 possible MDE cases (82.3%) remained. From this pool, participants that had missing depression symptom data were listwise deleted, leading to a final count of *n* = 5,749 participants (73.3%). In the NESARC, participants reported symptoms on their *worst depressive episode* within their *lifetime*. Thus symptom data were drawn retrospectively from episodes over the course of the participant’s lifetime.

### STAR*D

We also re-analyzed the Sequenced Treatment Alternatives to Relieve Depression (STAR*D; [13]). The STAR*D is a multi-site sequentially randomized clinical trial of 4,041 outpatients who were diagnosed with major depressive disorder (MDD). Inclusion criteria included being between the ages of 18 and 75 and a diagnosis of DSM-IV unipolar and non-psychotic MDD. Exclusion criteria included a history of mania or hypomania, schizophrenia, schizoaffective disorder or psychosis, or current anorexia, bulimia, or obsessive-compulsive disorder (OCD) as assessed by the Psychiatric Diagnostic Screening Questionnaire (PDSQ) via clinical interview [14]. Depressive symptoms, including melancholic and atypical symptoms, were screened using the Inventory of Depressive Symptomatology (IDS-SR). For more information regarding the study design, please refer to the following studies [4, 13]. The original sample had data available for 4,041 patients. Of these patients, 3744 (92.7%) provided baseline data during the first measurement point of the first treatment stage. We screened out patients who did not have full symptom-level IDS data, leading to 3,717 patients (91.9%). Inclusion criteria in the original trial required patients to meet the criteria for non-psychotic MDD based on a DSM-IV checklist. To ensure consistency, patients were screened for meeting an MDE based on the IDS itself, leading to *n* = 2,498 remaining patients (61.8%). Patients were queried on specific symptoms based on their *current* depressive episode. Thus, we derived diagnostic combinations from the STAR*D patients’ current depressive episode. See Lorenzo-Luaces et al. (2021; [8]) for a description of how the STAR*D symptoms were parsed.

### Outcomes

####  Alcohol Use Disorder and Associated Disabilities Interview Schedule (AUDADIS-IV)

In NESARC, the AUDADIS-IV [15] measures 19 symptoms of depression that are rated as either ‘present’ or ‘absent’ and coded as “1” or “2”, respectively. The AUDADIS-IV covers DSM-IV criteria symptoms in a disaggregated form. For example, it queries *both* psychomotor agitation and psychomotor retardation, whereas the DSM-IV codes psychomotor disturbances as a single symptom. In the end, we evaluated similarity across 16 symptoms. Below, we describe our decision-making process regarding symptom inclusion in the NESARC dataset.

##### Appetite or weight disturbances

The AUDADIS-IV contains four questions querying appetite or weight disturbances: 1) reduced appetite, 2) reduced weight, 3) increased appetite, and 4) increased weight. To prevent over-estimating the degree of heterogeneity in the data from overlapping symptoms, we combined the responses to the appetite and weight questions, thus creating two variables: 1) *decreased* appetite or weight and 2) *increased* appetite or weight. For decreased appetite/weight, we considered the person to have the symptom whether they reported decreased appetite, decreased weight, or both. Similarly, for increased appetite or weight, we considered the person to have the symptom whether they reported increased appetite, increased weight, or both.

##### Suicidal ideation

The AUDADIS-IV contains four questions pertaining to suicide: 1) death ideation (i.e., thoughts of death), 2) desire to die, 3) suicidal ideation (i.e., thoughts about killing oneself), and 4) attempted suicide. We distinguished suicidal attempts from thoughts by combining the responses to the first three questions (i.e., death ideation, desire to die, and suicidal ideation) into a symptom indicating the presence of suicidal thoughts. A person was considered to have suicidal thoughts if they expressed death ideation, desire to die, suicidal ideation, or some combination of these symptoms.

##### Restlessness and psychomotor agitation

The AUDADIS-IV queries an uncomfortable feeling of restlessness as well as symptoms of fidgeting and pacing as proxies for psychomotor agitation. We removed the ’feelings of restlessness’ symptom when performing the analyses, as subjective feelings of restlessness do not count towards the presence of psychomotor agitation per the DSM-5 (American Psychiatric Association, 2013).

##### Melancholic and atypical specifiers

The AUDADIS-IV does not query all the symptoms of melancholic and atypical depression. We categorized melancholic depression as having three symptoms from a list that included: anhedonia, psychomotor retardation/agitation, guilt, early morning awakenings, or significant weight loss. Comporting to previous NESARC analyses [16], the atypical subgroup consisted of respondents who met criteria for both hypersomnia and hyperphagia. The hierarchical rule of specifiers was also applied: Participants meeting criteria for a melancholic specifier could not then meet criteria for an atypical specifier (see appendix for a list of queried symptoms and criteria rules). The STAR*D dataset used the IDS to query for all depressive symptoms, including those for the melancholic and atypical specifiers. Thus, we adhered to the DSM-5’s criteria for melancholic and atypical specifiers in the STAR*D analyses.

### Analytic strategy

**Fig. 1:**
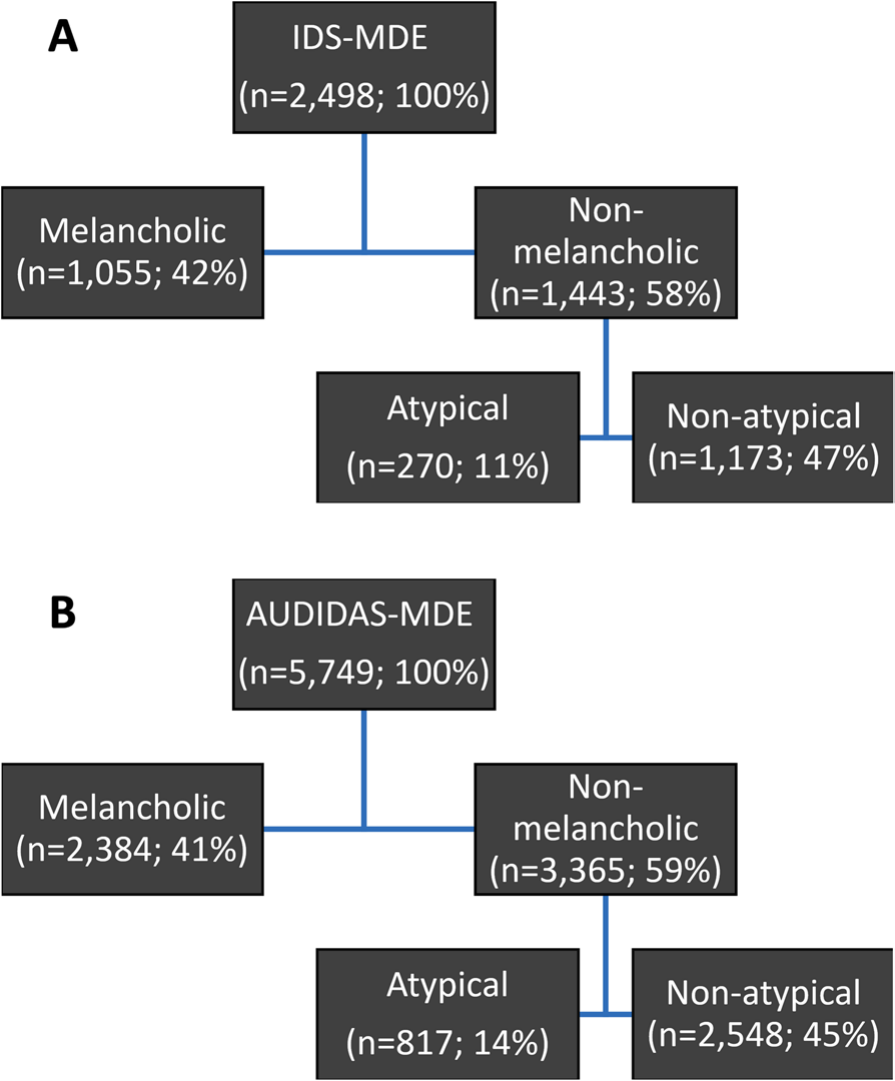
Melancholic and Atypical subgroups of patients derived from the IDS on the STAR*D (**A**) and AUDADIS-IV on the NESARC (**B**) datasets

Similar to previous analyses [8], we divided the NESARC and STAR*D datasets into subgroups corresponding to the presence of melancholic and atypical specifier subgroups, as shown in Fig. 1. Because we respected the hierarchical rule from DSM-5, all participants were screened for the presence of melancholia first, creating melancholic and non-melancholic subgroups. Then, all participants in the “non-melancholic” group were grouped into atypical vs. non-atypical subgroups.

All data were analyzed using the R programming language. All code is available at: https://osf.io/vh5qg/. Two functions calculating distance in N-dimensional space, known as the Hamming and Manhattan distances, were used [17, 18]. The Hamming formula is a way to measure distance in an N-dimensional space given two binary data strings (i.e., data containing only 0s and 1s). Equation 1a represents the formula for the Hamming distance (*D*_*H*_) for a dyad composed of person *x* and person *y*. *D*_*H*_ is calculated by summing the differences of two vectors in a vector space of symptoms represented by variable *k*, here representing the maximum number of possible symptoms. The term *x*_*i*_, represents symptom *i* within vector-space *k* of patient *x*, and *y*_*i*_ represents the same symptom *i* of patient *y*. For every specific symptom that is not shared between any two diagnostic combinations, the Hamming distance between the diagnostic combinations will increase by 1. Since the symptoms in NESARC were assessed as a binary, we used Hamming distances to calculate distances between individuals in their symptom endorsement.

**Equation 1:** Hamming Distance Function

1a $$\begin{aligned} D_H = \sum \limits _{i=1}^{k} |x_i - y_i| \end{aligned}$$

1b $$\begin{aligned} R_H = \frac{D_H}{||k||} \end{aligned}$$

Similar to the Hamming distance, the Manhattan distance quantifies the distance between two symptom vectors in an N-dimensional vector space *k*, which again refers to the total number of symptoms, as shown in Eq. 2a. The Manhattan distance for person *x* and person *y*, represented by *D*_*M*_, is calculated by summing the differences between two symptom profiles in a vector space of symptoms *k*, where *x*_*i*_ represents symptom *i* of patient *x*, and *y*_*i*_ represents the same symptom *i* of patient *y*. The Manhattan distance allows us to quantify distance in kind (i.e., symptom present vs. absent) as well as intensity (i.e., mild vs. severe presentations of the same symptom: see equation 2). A higher Manhattan distance between the diagnostic combinations of two individuals indicates a greater dissimilarity between them in the severity and kinds of symptoms. The Manhattan distance is not equivalent to a total sum score. Two combinations of symptoms can have equal total sum scores that arise from different symptom endorsements and would result in different Manhattan distances (see Appendix). Given that symptoms on the IDS were assessed on a polytomous 4-point scale, Manhattan distances were calculated for the STAR*D dataset.

**Equation 2:** Manhattan Distance Function

2a $$\begin{aligned} D_M = \sum \limits _{i=1}^{k} |x_i - y_i| \end{aligned}$$

2b $$\begin{aligned} R_M = \frac{D_M}{||k|| \cdot v} \end{aligned}$$

To simplify interpretation, all distance measures were standardized by dividing distance values by the length of the total possible symptom space. Equation 1b represents the standardized Hamming ratio score *R*_*H*_, where *D*_*H*_ is the calculated hamming distance, and the denominator is represented by the total number of symptoms queried or the maximum length of vector space *k*. Similarly, Eq. 2b displays the Manhattan ratio *R*_*M*_, which is calculated by dividing the total Manhattan distance *D*_*M*_ by the maximum length of vector space *k*. Because the Manhattan distance takes into account symptom severity we also divided by the scalar *v*, representing the maximum possible severity score. It should be noted that the STAR*D and NESARC datasets queried a different number of symptoms, thus the number of symptoms in vector space *k* differed between the two datasets.

Several separate sets of analyses were conducted. The first set of analyses used the NESARC dataset to calculate Hamming distances for each subgroup (i.e., melancholic vs. non-melancholic, and atypical vs. non-atypical) for the depressive symptoms present in the dataset for both within and between subgroups. We also calculated standardized Hamming and Manhattan distances in the STAR*D dataset. Given that the IDS assesses symptoms of depression as well as the symptoms of the specifiers, we conducted two additional sets of analyses. One that had all the symptoms of depression plus the specifiers, and another that only had the core DSM-5 symptoms of depression.

For each analysis, we calculated the within and between-group standardized distance. Within-subgroup calculations consisted of comparing each person in each diagnostic subgroup to each other person in that subgroup. For example, when evaluating Subgroup “A” (e.g., melancholic depression in STAR*D), the diagnostic combination of person C_a1_ was compared to the diagnostic combination of persons C_a2_, C_a3_, ... C_an_. Similarly, diagnostic combination C_a2_ was compared to C_a3_, C_a4_, ... C_an_. A distance metric was calculated between every other person only once within that subgroup and stored into a vector containing all calculated distances. Between-subgroup distance calculations compared each person’s symptom combination in a subgroup to each participant not meeting subgroup criteria (e.g., non-atypical profiles with atypical profiles). A standardized distance score was computed for each pairing and then stored into a vector containing all distances.

**Fig. 2:**
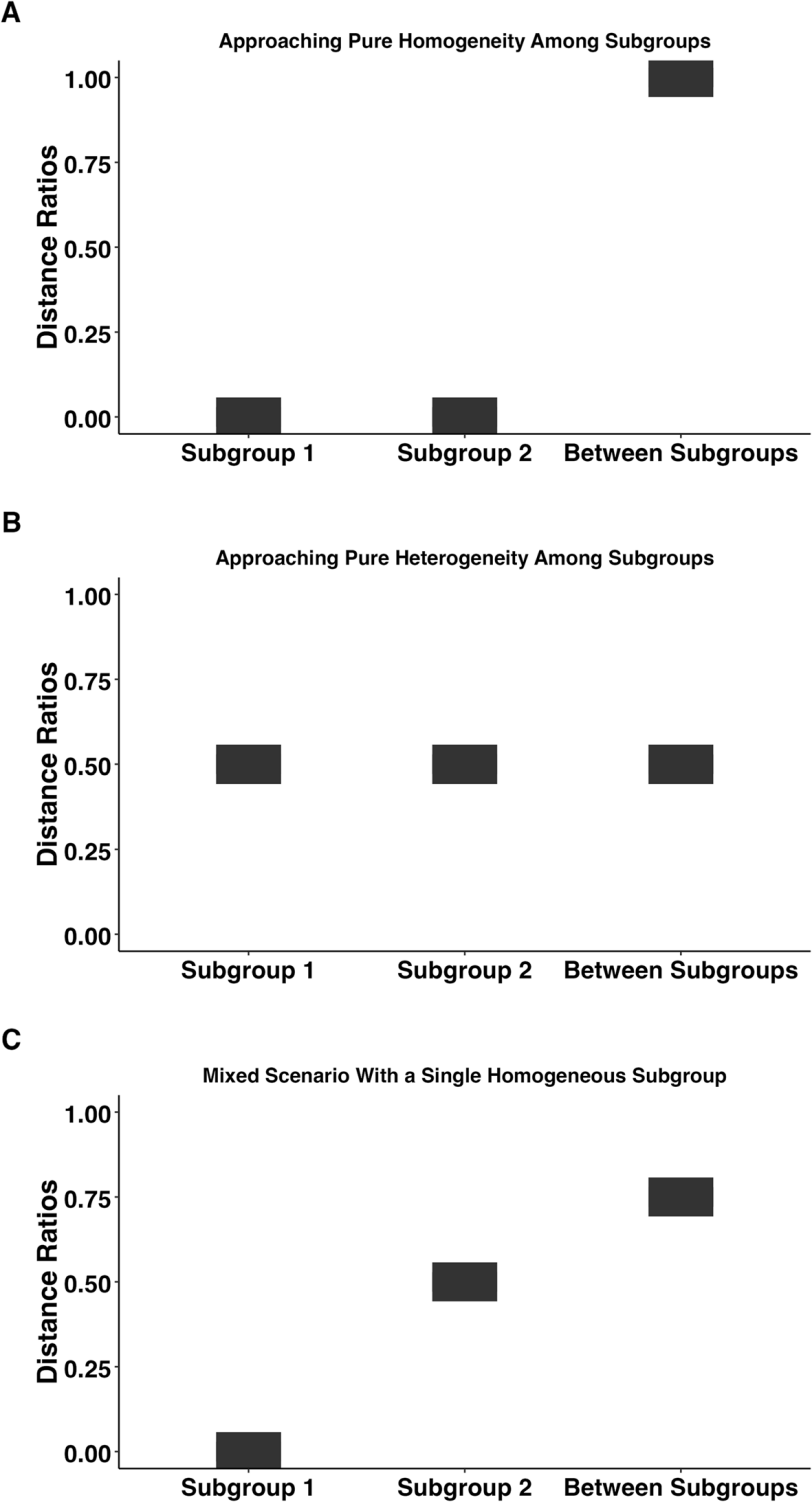
Illustration of distance ratios indicating ideal inner-group coherence and between-group differentiation between subgroup profiles, where subgrouping would be effective (**A**) heterogenous subgroup profiles generated using random data where subgrouping would be ineffective (**B**), and a mixed scenario where a homogeneous subgroup exists and subgrouping would be effective (**C**)

Due to the size of the datasets, the within-group and between-group vectors of distances comprised millions of data points. Thus, we illustrate all analyses using box plots to avoid data overcrowding. Three example boxplots are provided in Fig. 2, demonstrating how within-subgroup and between-subgroup analyses may be interpreted. Panel A shows an ideal case of subgroup coherence and differentiation (i.e., where subgroups show the maximal distance between diagnostic combinations). Subgroup 1 and Subgroup 2 are approaching pure coherence, as the distance ratios are 0; simultaneously, the two subgroups appear to be distinct, having high differentiation as the between-subgroup ratio approaches 1.

In contrast, Fig. 2 Panel B displays a case of complete heterogeneity. Both within- and between-subgroup analyses exhibit nearly identical distance ratios. The between-subgroup ratio indicates that the subgroups are low in differentiation (i.e., the diagnostic profiles in both subgroups are similar to each other), whereas the identical within-subgroup ratios indicate both subgroups are similarly heterogeneous.

Finally, in Fig. 2 Panel C, we show a “mixed” scenario where the specifier groups could capture a homogeneous subgroup of patients (with distance scores of 0), but the non-specifier group is still heterogeneous (e.g., with distance scores around 0.5). In such a scenario, we would still see large between-group distances (0.75) and this would be an indication of the specifier reducing diagnostic heterogeneity. Indeed, this example would be more likely the case than Fig. 2 Panel A, if these specifiers were creating more coherent subgroups.

To rule out the possibility that the differences observed between specifier groups could be accounted for by chance, we conducted a series of permutation tests as in our previous study [8] to test whether the between-group differences were above and beyond what would be expected by chance. We conducted the permutation tests by randomly shuffling the specifier and non-specifier labels, obtaining a random dyad, and then obtaining distance scores for that dyad. We repeated this process 100 times for each between-group distance we present (i.e., for each specifier, for each dataset, for each set of symptoms). The *p*-values represent the probability that one would obtain a between-group distance as or more extreme than the one we observed by random chance.

## Results

### Symptom endorsement

Table 1 shows the descriptive statistics representing the binary endorsement of symptoms (i.e., yes vs. no) criteria for a DSM-IV MDE within the NESARC dataset. A table of symptom endorsement for the STAR*D dataset representing the presence or absence of symptoms in the patients meeting criteria for an IDS-MDE can be found in the Appendix. In NESARC, sad mood and (94.97%) and anhedonia (87.60%) were the most frequently reported symptoms. The least endorsed symptoms were suicide attempt (11.05%) and appetite/weight increase (36.42%).

Of the individuals in the subset of the NESARC data we used, 2,384 (41.47%) met criteria for melancholic depression, and 3,365 (58.53%) met criteria for non-melancholic depression. Whereas 817 (14.21%) met criteria for atypical depression, and the remainder 2,548 (44.32%) met criteria for non-atypical depression. The proportion of participants in the melancholic and atypical NESARC specifier subgroups are similar to the specifier frequencies in the STAR*D dataset: melancholic (42.23%), non-melancholic (57.77%), atypical (10.81%), and non-atypical (46.96%).

**Table 1:** Endorsement of specific symptoms of DSM criteria for major depression, melancholia, and atypical specifiers in patients with MDD, MDD with melancholia features, and MDD with atypical features, as determined by the AUDADIS-IV

	AUDADIS-MDE		AUDADIS-Mel		AUDADIS-Aty	
Symptom	%	(n)	%	(n)	%	(n)
Sad mood	94.97	5460	95.51	2277	94.12	769
Anhedonia	87.60	5036	100	2384	85.68	700
Appetite/weight decrease^a^	60.08	3454	86.45	2061	17.87	146
Appetite/weight increase^b^	36.42	2094	29.07	693	100	817
Insomnia sleep onset	69.42	3991	84.02	2003	46.88	383
Early morning awakening	54.90	3156	82.38	1964	21.67	177
Hypersomnia^b^	46.83	2692	40.86	974	100	817
Psychomotor retardation^a^	40.76	2343	61.37	1463	30.23	247
Psychomotor agitation^a^	37.50	2156	60.74	1448	20.20	165
Fatigue	84.71	4870	84.94	2025	92.66	757
Worthlessness	62.55	3596	74.20	1769	59.73	488
Guilt^a^	58.13	3342	81.92	1953	47.49	388
Diminished concentration	84.71	4870	91.99	2193	80.78	660
Indecisiveness	75.77	4356	85.19	2031	71.60	585
Suicidal ideation/Thoughts of dying	59.51	3421	65.18	1554	56.55	462
Suicide attempt	11.05	635	14.60	348	8.94	73

^a^ also a symptom of the ‘melancholic features’ specifier,

^b^ also a symptom of the ‘atypical features’ specifier

**Fig. 3:**
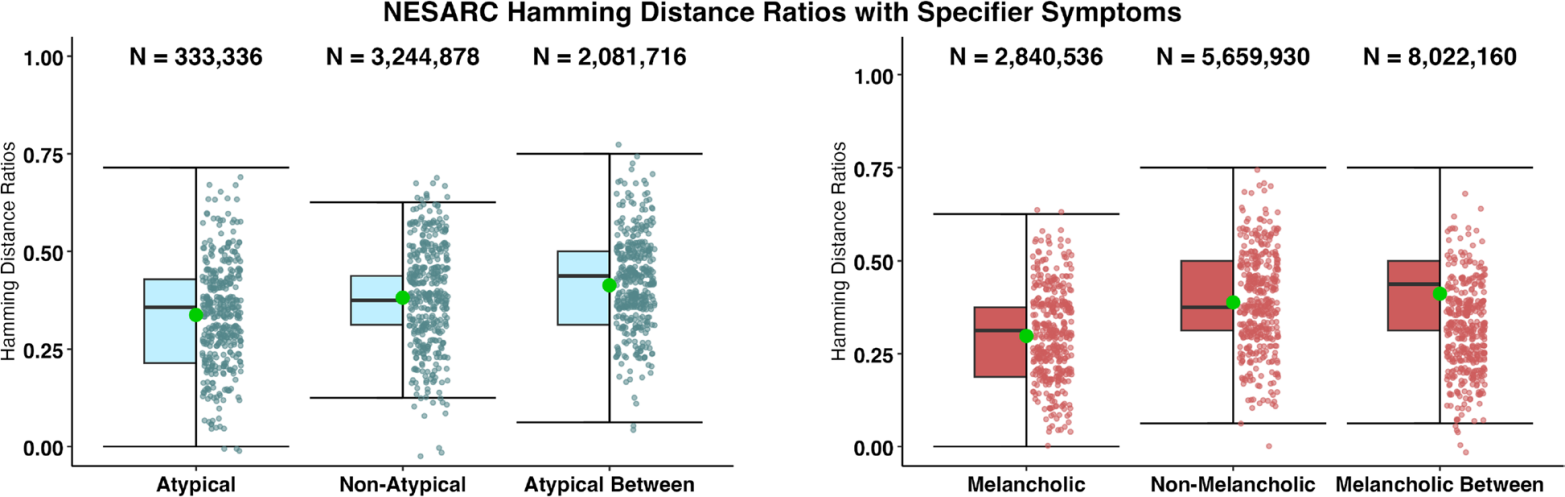
Hamming distance ratios of diagnostic combinations for patients meeting MDD criteria queried by the AUDADIS-IV within the NESARC dataset

### NESARC

The Hamming distances can be found in Fig. 3. The results of our analyses in multivariate space within the melancholic and non-melancholic subgroups, as well as between melancholic and non-melancholic subgroups, suggest that this specifier does not increase coherence. The melancholic and non-melancholic subgroups show similar median Hamming distance ratios (Med_mel_ = 0.333, IQR_mel_ = [0.200, 0.400]) and Med_non-mel_ = 0.375, IQR_non-mel_ = [0.313, 0.500]). When comparing the within group coherence for each specifier group to the within-group for the entire MDD sample, we see negligible differences between medians (Med_MDD_ = 0.375, IQR_MDD_ = [0.313, 0.500]). While the between-groups median Hamming ratio (Med_btw-mel_ = 0.438, IQR_btw-mel_ = [0.313, 0.500]) was also close to the within-group Hamming ratios, indicating low differentiation between the melancholic and non-melancholic subgroups. Figure 3 appears to resemble Fig. 2 Panel B, whose data were generated at random. These findings suggest that the subgroups are not meaningfully different when only looking at symptom heterogeneity of diagnostic combinations. The permutation test for the melancholic and non-melancholic group was not significant (*p* = 0.49) suggesting the between-group distances are not greater than would be expected by chance.

Similar to the melancholic and non-melancholic subgroups, the atypical and non-atypical subgroups show similar median Hamming distance ratios (Med_aty_ = 0.357, IQR_aty_ = [0.214, 0.429] and Med_non-aty_ = 0.375, IQR_non-aty_ = [0.313, 0.438]), indicating few differences of within-subgroup coherence. While the between subgroups median Hamming ratio (Med_btw-aty_ = 0.438, IQR_btw-aty_ = [0.313, 0.500]) is close to the within-subgroup Hamming ratios, indicating low differentiation between the atypical and non-atypical subgroups. The results of the permutation tests comparing the atypical and non-atypical between group distances with a distribution of randomly generated group scores was non-significant (p = 0.48) suggesting the groups do not differ more than would be expected by chance.

**Fig. 4:**
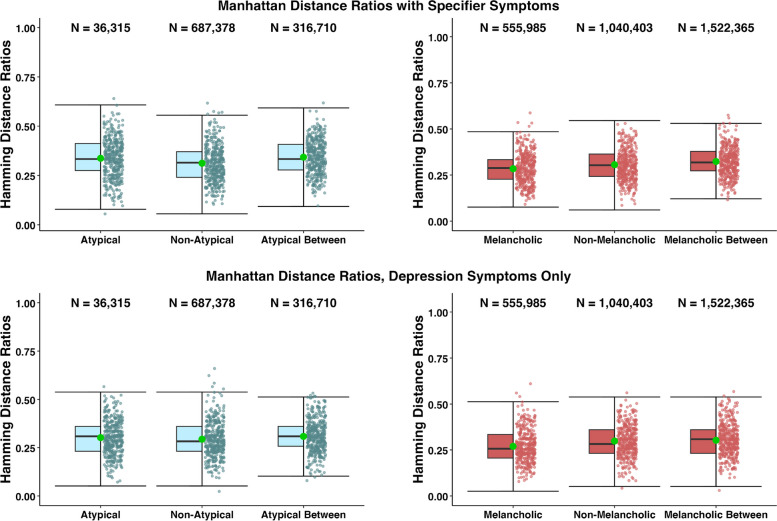
Manhattan distance ratios of diagnostic combinations for patients meeting MDD criteria queried by the IDS-SR within the STAR*D dataset, with specifier symptoms, and without specifier symptoms

### STAR*D

We created boxplots to represent the within- and between-subgroup distances in multivariate space using the STAR*D dataset. The same sets of analyses were performed using Manhattan distances, where we allowed each symptom to vary on a 4-point scale (i.e., 0-3). Boxplots of the STAR*D Manhattan distances can be found in Fig. 4. The STAR*D melancholic and non-melancholic subgroups displayed similar levels of distance in multivariate space within-subgroup (Med_mel_ = 0.288, IQR_mel_ = [0.227, 0.333], Med_non-mel_ = 0.303, IQR_non-mel_ = [0.242, 0.364]) and when comparing between-subgroup (Med_mel-btw_ = 0.318, IQR_mel-btw_ = [0.273, 0.379]), suggesting the melancholic specifier does not increase subgroup coherence. The permutation test for the melancholic and non-melancholic groups was non-significant (p = 0.79), indicating the groups do not differ more than would be expected by chance.

The within and between-subgroup comparisons in atypical vs. non-atypical depression suggested the atypical specifier does not increase coherence (Med_aty_ = 0.333, IQR_aty_ = [0.275, 0.412], Med_non-aty_ = 0.315, IQR_non-aty_ = [0.241, 0.370], and Med_aty-btw_ = 0.333, IQR_aty-btw_ = [0.278, 0.408]). Finally, the results of the permutation tests for the STAR*D dataset comparing the atypical and non-atypical between group Manhattan scores with a distribution of permuted group scores were non-significant (p =0.67). Additionally, comparing the within-group coherence for each specifier group to the within-group for the entire MDD sample, we see negligible differences between medians (Med_MDD_ = 0.364, IQR_MDD_ = [0.273, 0.409]).

When only focusing on the core DSM-5 depressive symptoms (i.e., ignoring the specifier symptoms), neither the melancholic (Med_mel_ = 0.256, IQR_mel_ = [0.205, 0.333], Med_non-mel_ = 0.282, IQR_non-mel_ = [0.231, 0.359], and Med_mel-btw_ = 0.308, IQR_mel-btw_ = [0.231, 0.359]) nor the atypical specifier appeared to increase coherence (Med_aty_ = 0.308, IQR_aty_ = [0.231, 0.359], Med_non-aty_ = 0.282, IQR_non-aty_ = [0.231, 0.356], and Med_aty-btw_ = 0.308, IQR_aty-btw_ = [0.256, 0.359]; see Fig. 4).

## Discussion

We examined whether the melancholic and atypical specifiers for MDD reduce symptom heterogeneity. Across a nationally representative and a clinical sample, for each participant’s symptoms, we computed distance metrics relative to the symptoms of other individuals in the samples, as measures of heterogeneity. Consistent with prior work, our results did not suggest that specifiers reduce diagnostic heterogeneity. We suggest the use of distance metrics for quantifying symptom heterogeneity over traditional symptom profile category methods. The distance metrics may be somewhat less intuitive to understand. However, they provide more explanatory power in that they calculate heterogeneity on a continuum rather than on a binary (i.e., people are alike or they are not).

Our primary objective in using Hamming and Manhattan distance metrics was to quantify symptom heterogeneity at a given timepoint. It is, however, important to emphasize that the Manhattan and Hamming distances are not intended for clinical assessment. They are also not intended to supplant sum scores from depression. Sum scores are an efficient measure of severity and have shown predictive validity [19–21]. Like the proponents of latent variable models of psychopathology and the proponents of network theory, we are concerned about the level of symptom heterogeneity in DSM diagnoses. The distance metrics measure heterogeneity; however, the distance metrics are not meant to capture theoretical relationships among the symptoms, including either latent dimensions or networks of interrelated symptoms. 

There are many types of distance functions that could be used to quantify distance across profiles (e.g., Euclidean, cosine, Minkowski distance). We chose the Manhattan and Hamming distances due to their relative intuitiveness. For example, if any two patients differed in only two symptoms and both by a severity of three, the Manhattan distance would add up to six, while other popular distance functions such as the Euclidean distance would be $3\sqrt{2}$, or 4.2. We find that a whole number (e.g.,six) is more easily interpretable, without sacrificing information, than a decimal (e.g., $3\sqrt{2}$). Furthermore, there is evidence that Manhattan distances may be preferable when there are high levels of dimensionality (e.g., multiple symptoms) in the data [22].

### Strengths and limitations

Several limitations of the current analysis are worth considering. First, patients were excluded from the STAR*D dataset if they reported psychosis, met criteria for anorexia, bulimia, substance dependence, primary OCD, or had prior non-response to citalopram. The only exclusion criteria applied to the NESARC dataset were a lifetime history of mania and hypomania and an illness or a substance-induced MDE. Thus the current results may not generalize to patients with bipolar depression, medication-induced depression, and depression due to a general medical condition. Secondly, NESARC did not query for all additional specifier symptoms required for the atypical and melancholic criteria, thus we used proxy definitions for these specifiers. Although prior studies have used proxy definitions and found these proxies valid for the melancholic and atypical specifiers, there may have been misclassifications relative to relying on the DSM. Third, our results do not indicate whether melancholic and atypical subgroups are valid clinical constructs that “carve nature at its joints,” nor do our results inform whether they are useful in terms of predicting metrics of interest (e.g., treatment outcomes). Finally, we did not examine whether the specifier subgroups are biologically-homogeneous constructs (e.g., as indexed by biomarkers).

Despite these limitations, our study has notable strengths. First, we tested a long-standing assumption of the DSM: that specifier subgroups reduce heterogeneity. Second, we used two large and well-characterized samples that complemented each other. Finally, we moved beyond prior work that has relied on counting symptom profile categories without quantifying heterogeneity between individuals with continuous metrics. Prior analyses have used metrics requiring 100% agreement in all symptoms to count individuals as being homogeneous. Depression and other forms of psychopathology appear to be better characterized by a continuum of severity rather than a categorical labels, at least between individuals [11]. Thus, heterogeneity between individuals, may be better represented on a continuum rather than categorically (i.e., same profile vs. not the same profile).

### Implications

Developing valid specifiers for psychopathology may have many benefits, including 1) elucidating specific etiologic mechanisms, 2) creating prescriptive categories that may be used by treatment-matching algorithms, 3) identifying clinical phenomena (e.g., risk factors, prognosis), 4) and creating more coherent subgroups of patients. However, our results do not indicate that DSM-5 atypical and melancholic specifiers create more coherent subgroups of patients. Although the melancholic and atypical subtypes have been long-rooted in historical contexts and preserved through the editions of the DSM, the evidence supporting their construct validity is weak, and there is inconsistent evidence of their biological correlates [23–25]. Additionally, there is a dearth of studies supporting the predictive validity of the melancholic and atypical subtypes, at least in matching to cognitive-behavioral therapy vs. SSRIs [23, 26–28].

Moreover, the current DSM’s definitions of the atypical and melancholic features may not accurately capture the intended subgroups. In the case of melancholia, for example, a significant divergence in defining the construct between researchers and the DSM-5 is apparent [29, 30]. Many proponents claim psychomotor retardation, and mood non-reactivity are the main components of melancholia [31] whereas an endogenous onset of depression has also been raised as melancholia's hallmark feature [30, 32]. One avenue for future work may be to propose theoretical accounts of melancholic or atypical depression [33], specifying whether they are better understood as networks of reinforcing symptoms, interactions of latent vulnerabilities (e.g., thought disorder X psychomotor disturbances X detachment), or clusters of symptoms that are differentially aggregated across people. Alternatively, specific symptoms themselves may indicate more coherent subgroups. For example, both positive affectivity and sleep disturbances appear significant in predicting symptom change during treatment and may be suitable candidate endophenotypes to pursue [34–37]. Indeed, taking a symptoms-based approach may help disassemble the potentially relevant biomarkers of the melancholic and atypical subtypes. For example, elevated cortisol levels in the morning may indicate the presence of melancholia or they may indicate the melancholic symptom ‘early morning awakening’.

Research on potential depressive subtypes appears to assume a latent variable model rather than an alternative model like a network-focused approach [38–40]. Researchers have shown that individual depressive symptoms have differing heritability [41] and correlate differentially with clinical validators (e.g., prognosis, comorbidities; [42]). Further, there is a burgeoning discussion surrounding the etiologies and biological mechanisms associated with specific symptoms. Researchers have proposed that neurovegetative depressive symptoms (e.g., sleep disturbances, psychomotor changes) but not cognitive symptoms (e.g., impaired concentration) have strong associations with inflammation biomarkers, thus suggesting differential etiologic pathways to specific symptoms [43]. Diagnostic heterogeneity, thus, may correspond to etiological heterogeneity, but it is necessary to clarify the differences between heterogeneity in symptoms vs. heterogeneity in causal pathways. Given the push to identify new depressive endophenotypes, the use of more refined diagnostic heterogeneity measures, such as the one we employed, should be considered when making comparisons between diagnostic categories or systems. In other words researchers should test, and not just assume, that specific subgroups reduce heterogeneity.

## Conclusion

The current study does not support the claim that melancholic and atypical depressive specifiers reduce diagnostic heterogeneity, as operationalized by distance metrics. The use of distance functions may prove valuable when assessing the utility of psychopathology’s current and future diagnostic systems. Future research should further assess the utility of heterogeneity metrics and other potential measures for quantifying symptom heterogeneity, severity, and symptom development over time.

## Data Availability

The data that support the findings of this study are openly available in NIMH data archive, https://nda.nih.gov/. The code for the analyses can be found here: https://osf.io/v8sbe/.
